# Doublet or Triplet Antiemetic Prophylaxis for Nausea and Vomiting Induced by Trastuzumab Deruxtecan: an Open-Label, Randomized, and Multicenter Exploratory Phase 2 Study

**DOI:** 10.7150/jca.87169

**Published:** 2023-08-28

**Authors:** Hirotoshi Iihara, Mototsugu Shimokawa, Hiroko Bando, Yoshimi Niwa, Yutaka Mizuno, Yoshihiro Kawaguchi, Mika Kitahora, Akari Murakami, Masaaki Kawai, Kazushige Ishida, Makoto Takeuchi, Kazuhiro Ishihara, Tomokazu Iyoda, Takumi Nakada, Atsuko Ogiso, Yasuyuki Kojima, Fumiyoshi Kumagai, Aya Sawa, Ryutaro Mori, Kosuke Higuchi, Tomoko Furuta, Yoshiaki Kamei, Masami Tsuchiya, Azusa Terasaki, Senri Yamamoto, Mai Kitazawa, Mai Okazaki, Akio Suzuki, Manabu Futamura

**Affiliations:** 1 Department of Pharmacy, Gifu University Hospital, 1-1 Yanagido, Gifu, Gifu 501-1194, Japan.; 2 Patient Safety Division, Gifu University Hospital, 1-1 Yanagido, Gifu, Gifu 501-1194, Japan.; 3 Laboratory of Pharmacy Practice and Social Science, Gifu Pharmaceutical University, 1-25-4 Daigakunishi, Gifu, Gifu 501-1196, Japan.; 4 Department of Biostatistics, Yamaguchi University Graduate School of Medicine, 1-1-1 Minamikogushi, Ube, Yamaguchi 755-8505, Japan.; 5 Cancer Biostatistics Laboratory, Clinical Research Institute, National Hospital Organization Kyushu Cancer Center, 3-1-1 Notame, Minami-ku, Fukuoka 811-1395, Japan.; 6 Institute of Medicine, Breast and Endocrine Surgery, University of Tsukuba, 1-1-1 Tennodai, Tsukuba, Ibaraki 305-8575, Japan.; 7 Department of Breast Surgery, Gifu University Hospital, 1-1 Yanagido, Gifu, Gifu 501-1194, Japan.; 8 Department of Breast Surgery, Yokkaichi Municipal Hospital, 2-2-37 Shibata, Yokkaichi, Mie 510-8567, Japan.; 9 Department of Breast Surgery, Asahi University Hospital, 3-23 Hashimoto-cho, Gifu, Gifu 500-8523, Japan.; 10 Department of Breast Center, Ehime University Hospital, 454 Shitsukawa, Toon, Ehime 791-0295, Japan.; 11 Department of Breast Surgery, Miyagi Cancer Center, 47-1 Aza-nodayama, Medeshima-shiote, Natori, Miyagi 981-1239, Japan.; 12 Department of First Surgery, Yamagata University Graduate School of Medicine, 2-2-2 Iida-Nishi, Yamagata, Yamagata, 990-9585, Japan (present address).; 13 Department of Surgery, Iwate Medical University, 2-1-1 Idaidori, Yahaba, Shiwa, Iwate 028-3695, Japan.; 14 Department of Breast Surgery, Central Japan International Medical Hospital, 1-1 Kenkonomachi, Minokamo, Gifu 505-8510, Japan.; 15 Department of Surgery, Gihoku Kosei Hospital, 1187-3 Takatomi, Yamagata, Gifu 501-2105, Japan.; 16 Department of Pharmacy, Fukushima Medical University Hospital, 1 Hikariga-oka, Fukushima 960-1247, Japan.; 17 Department of Breast Surgery, Gifu Municipal hospital, 7-1 Kashimacho, Gifu, Gifu 500-8513, Japan.; 18 Department of Breast Surgery, Gifu Prefectural General Medical Center, 4-6-1 Noishiki, Gifu, Gifu 500-8717, Japan.; 19 Department of Breast and Endocrine Surgery, St. Marianna University School of Medicine, 2-16-1 Sugao, Miyamae, Kawasaki, Kanagawa 216-8511, Japan.; 20 Department of Pharmacy, Tohoku Rosai Hospital, 4-3-21 Dainohara, Miyagino, Sendai, Miyagi 981-8563, Japan.; 21 Department of breast, thyroid, endocrine surgery, University of Tsukuba Hospital, 2-1-1 Amakubo, Tsukuba, Ibaraki 305-8576, Japan.; 22 Department of Pharmacy, Yokkaichi Municipal Hospital, 2-2-37 Shibata, Yokkaichi, Mie 510-8567, Japan.; 23 Department of Pharmacy, Asahi University Hospital, 3-23 Hashimoto-cho, Gifu, Gifu 500-8523, Japan.; 24 Department of Pharmacy, Miyagi Cancer Center, 47-1 Nodayama, Medeshimashiote, Natori, Miyagi 981-1293, Japan.; 25 Laboratory of Advanced Medical Pharmacy, Gifu Pharmaceutical University, 1-25-4 Daigakunishi, Gifu, Gifu, 501-1196, Japan.

**Keywords:** breast cancer, antiemetic regimen, trastuzumab deruxtecan, nausea, vomiting

## Abstract

**Background:** Trastuzumab deruxtecan is classified as an anticancer agent that poses a moderate emetic risk in the international guidelines for antiemetic therapy. The guidelines recommend emesis prophylaxis using a two-drug combination therapy comprising a 5-hydroxytryptamine-3 receptor antagonist (5-HT3RA) and dexamethasone (DEX). However, the high incidence of nausea and vomiting associated with trastuzumab deruxtecan is problematic. The National Comprehensive Cancer Network guideline version 1.2023 classified trastuzumab deruxtecan as having a high risk of emesis and changed its recommendation to a triplet regimen including a neurokinin-1 receptor antagonist (NK1RA). However, the emetogenic potential of trastuzumab-deruxtecan and the optimal antiemetic prophylaxis are controversial. Hence, this exploratory phase 2 study aimed to assess the efficacy and safety of treatment comprising 5-HT3RA and DEX with or without a NK1RA in preventing trastuzumab deruxtecan-induced nausea and vomiting.

**Methods:** We conducted an open-label and randomized exploratory phase 2 study at 14 centers in Japan. Patients with breast cancer who were scheduled to receive trastuzumab deruxtecan were enrolled in this study. The patients were randomly assigned to receive granisetron and DEX (arm GD) or granisetron, DEX, and aprepitant (fosaprepitant; arm GDA). The primary endpoint was complete response (CR; no emesis or no rescue therapy) during the overall phase (120 h after the start of trastuzumab deruxtecan).

**Results:** Between September 2020 and March 2023, 40 patients were randomly assigned to the GD (n = 19) or GDA (n = 21) arm. In the GDA arm, one patient who did not complete the use of the rescue medication listed in the diary was excluded from the efficacy analysis, which included the use of rescue medication. The CR rates during the overall phase were 36.8% and 70.0% in the GD and GDA arms, respectively (odds ratio 0.1334; 95% confidence interval [CI]: 0.0232-0.7672; P = 0.0190), with a difference of 33.2%. No grade 3 or 4 toxicity related to antiemetic therapy was observed.

**Conclusions:** Patients receiving trastuzumab deruxtecan require triple therapy, including mandatory NK1RA administration.

## Introduction

Trastuzumab deruxtecan is a conjugate of the humanized anti-human epidermal growth factor receptor 2 (HER2) antibody with topoisomerase I inhibitor payload using an enzymatically cleavable peptide-based linker 1. The cytotoxic payload is deruxtecan, which is a camptothecin analog. Trastuzumab deruxtecan has been developed for the treatment of HER2-expressing solid tumors 2,3. In the DESTINY-Breast01 trial, trastuzumab deruxtecan showed an extremely high level of clinical activity with an overall response rate of 60.9% and progression-free survival of 16.4 months in a previously treated patient population with HER2-positive metastatic breast cancer 4. On December 20, 2019, the drug received an accelerated approval from the United States Food and Drug Administration for treating patients with unresectable or metastatic HER2-positive breast cancer. It has since been approved for use in Japan and Europe. Trastuzumab deruxtecan is highly effective against HER2-positive gastric cancer, HER2-mutated non-small-cell lung cancer, and HER2-expressing metastatic colorectal cancer 5-7. However, it was associated with an elevated rate of nausea and vomiting in the corresponding trials. The respective incidence rates of nausea and vomiting in patients with breast, gastric, non-small cell lung, and colorectal cancers were as follows: 77.7% and 45.7%, 63% and 26%, 73% and 40%, and 62.3% and 29.5% 5-7. In response, the American Society of Clinical Oncology guideline for antiemetics and the National Comprehensive Cancer Network (NCCN) Clinical Practice Guidelines in Oncology (Antiemesis version 1.2020), classified trastuzumab deruxtecan as an anticancer agent with a moderate emetic risk 8,9. However, the DESTINY trials did not explore antiemetic therapy or the antiemetic effects against trastuzumab deruxtecan action. Moreover, there is no information available in terms of antiemetic therapy. DESTINY-Breast04 study, the protocol recommended prophylactic antiemetic therapy with a 5-hydroxytryptamine receptor antagonist (5-HT3RA) or neurokinin-1 receptor antagonist (NK1RA) and/or a steroid, such as dexamethasone (DEX) since 2020. However, it is unclear which combination regimen was most often administered. 10.

Recent guidelines recommend emesis prophylaxis using a two-drug combination therapy comprising a 5-HT3RA and DEX for chemotherapy with a moderate emetic risk 8,9,11,12. However, these guidelines suggest a three-drug combination therapy comprising a 5-HT3RA, DEX, and NK1RA prophylactic regimen for patients with additional risk factors or those in whom previous treatment with a 5HT3RA and DEX failed 9. There are also anticancer agents, such as carboplatin, that are considered highly emetogenic with a moderate emetic risk (classified as high emetic risk by the NCCN) but are recommended as a special exception for triplet therapy comprising 5-HT3RA, DEX, and NK1RA. In January 2023, the NCCN guidelines (version 1.2023) reclassified trastuzumab deruxtecan as having a high emetic risk 9.

It is unclear whether two- or three-drug combinations should be recommended for trastuzumab deruxtecan-treated patients with a high incidence of nausea and vomiting. Complete response (CR) is a standard method of evaluating antiemetic therapy. This study aimed to investigate the CR (no emesis or rescue therapy) rate of trastuzumab deruxtecan-treated patients with breast cancer using a two-drug combination of granisetron and DEX or a three-drug combination of granisetron, DEX, and aprepitant (fosaprepitant) as antiemetic treatments.

## Methods

### Study design

This open-label, multicenter, and randomized exploratory phase 2 study was conducted in 14 Japanese hospitals (cancer centers, private hospitals, public hospitals, and university hospitals) in accordance with the Declaration of Helsinki and the Ethical Guidelines for Clinical Studies. The study was approved by the institutional review board of each participating hospital and independently monitored by the Alliance Data Center and Safety Monitoring Board. Data collection and analysis were conducted using alliance statistics and data centers. Data quality was ensured by a review of the data performed by the Alliance Statistics and Data Center and by the principal investigator according to Alliance policies. The study was registered with the University Hospital Medical Information Network (UMIN000041004; principal investigators: HI and MF; primary statistician: MS).

### Randomization

Eligible patients were randomized (1:1) to receive either granisetron and DEX (GD arm) or granisetron, DEX, and aprepitant/fosaprepitant (GDA arm). Registration and randomization were performed using a web entry system requiring a personal account and password. The participants were stratified and randomly allocated to either the GD or GDA arm by using permuted blocks. The stratification was based on two factors, age (≥ 55 years vs < 55 years) and previous experience with chemotherapy-induced nausea and vomiting (CINV; yes vs no). The block sizes were set to 2 and 4 to guarantee balanced allocation.

### Patient selection

Patients with breast cancer scheduled to receive trastuzumab deruxtecan for the first time were enrolled in this study. Other eligibility criteria were as follows: age of ≥ 20 years; absence of current use of any drug with antiemetic activity or drugs with a risk of nausea, for example, 5-HT3RA, NK1RA, corticosteroids, antidopamine agonists, phenothiazine tranquilizers, serotonin dopamine antagonists, multi-acting receptor-targeted antipsychotics, benzodiazepine agents, selective serotonin reuptake inhibitors, and serotonin noradrenaline reuptake inhibitors; aspartate aminotransferase and alanine aminotransferase levels of ≤ 100U/L; total bilirubin level of ≤ 2.0 mg/dL; and provision of written informed consent.

The exclusion criteria were as follows: patients with a history of hypersensitivity or allergy due to the study drugs or similar compounds; patients who needed antiemetics at enrollment; patients who started taking opioids within 48 h before enrollment; patients with unstable angina, ischemic heart disease, cerebral hemorrhage or apoplexy, or an active gastric or duodenal ulcer within 6 months before enrollment; patients with convulsive disorders requiring anticonvulsant therapy; patients with ascites effusion requiring paracentesis; patients with gastrointestinal obstruction; breastfeeding or expecting women or those who did not wish to use contraception; patients with psychosis or psychiatric symptoms that interfere with daily life; and patients who were judged to be unsuitable for the study by the investigators.

### Treatment regimen

Antiemetic therapy was administered according to the ASCO 2020 recommendations for carboplatin as follows 8: patients in both treatment arms were administered granisetron (1 mg intravenous infusion 30 min before trastuzumab deruxtecan on day 1). Those in the GD arm were administered DEX (6.6 mg intravenous infusion 30 min before trastuzumab deruxtecan administration on day 1 and 8 mg oral administration on days 2-3). The patients in the GDA arm were administered DEX (9.9 mg intravenous infusion 30 min before trastuzumab deruxtecan on day 1) and NK1RA (125 mg of aprepitant orally administered 60 min before chemotherapy on day 1 and 80 mg oral administration on days 2 and 3, or 150 mg fosaprepitant intravenous infusion 60 min before trastuzumab deruxtecan on day 1). Patients were allowed to take rescue medication for CINV, if necessary, throughout the study period. All the patients were prescribed rescue medications. The choice of the recommended rescue medication was determined from among metoclopramide, domperidone, and prochlorperazine specified in the protocol by each investigator.

### Assessment procedures

All pertinent demographic characteristics and medical data were recorded during the pre-study period. Patients' physical examinations and blood tests were scheduled before treatment initiation. Data were collected from the patient diaries. Patients filled out the diary every 24 h from the start of trastuzumab deruxtecan treatment until 168 h, in which they reported daily the presence of nausea and decreased appetite by using a four-item scale on which the symptom severity was rated as none, mild, moderate, and severe. The frequency of vomiting was reported using a five-item scale as follows: 0 times, 1-2 times, 3-5 times, 6 times or more, and almost always. The use of rescue medication was reported using a four-item scale as follows: 0 times, 1 time, 2 times, and 3 times or more. In addition, the following items of the Patient Reported Outcome (PRO) Common Terminology Criteria for Adverse Events (CTCAE) version 1.0. were reported after the extended-overall phase (0-168 h): nausea, vomiting, decreased appetite, taste changes, hiccups, constipation, and diarrhea. To obtain baseline data, an assessment was performed before the start of trastuzumab deruxtecan therapy. Patient satisfaction with antiemetic therapy was measured using a seven-grade scale (very satisfied, satisfied, somewhat satisfied, rather satisfied, rather dissatisfied, dissatisfied, and very dissatisfied) after the extended-overall phase. The CINV assessment was performed on the basis of the patient's diary.

### Outcomes

The primary endpoint was the CR rate during the overall phase (0-120 h) after the initiation of trastuzumab deruxtecan therapy. The secondary endpoints were the CR rates during the extended-overall phase (0-168 h), acute phase (0-24 h), delayed phase (24-120 h), and extended-delayed phase (24-168 h) and the complete control (CC) rate, defined as no emetic episode; no rescue medication use; and no significant nausea during the acute, delayed, extended-delayed, overall phase, and extended-overall phases. Significant nausea was defined as any rating greater than “mild nausea” on the four-point scale. The total control (TC) rate was defined as no emetic episodes, no rescue medication use, and no nausea during the acute, delayed, extended-delayed, overall phase, and extended-overall phases. The rates of nausea and vomiting were assessed during the acute, delayed, extended-delayed, overall, and extended-overall phases. Time to treatment failure (TTF) was defined as the time from the initiation of trastuzumab deruxtecan therapy to the first episode of vomiting or to rescue medication use, whichever happened first; and patient satisfaction with antiemetic therapy. Adverse events were graded according to PRO-CTCAE version 1.0 and CTCAE version 5.0.

### Statistical analysis

This exploratory phase 2 study aimed to determine the optimal antiemetic therapy for trastuzumab deruxtecan-treated patients with breast cancer. To date, there is no knowledge about the CR rate during the 5-day phase (within 120 h of the start of trastuzumab deruxtecan administration), which is the primary endpoint. Therefore, because the number of cases could not be based on a statistical hypothesis, the number of cases that could be evaluated clinically with minimal accuracy against the obtained results was set (GD arm: 20 cases; GDA arm: 20 cases) by considering the number of cases that could be accumulated within the enrollment phase.

The primary endpoint was estimated using the CR rate and 95% confidence intervals (CIs) according to the Pearson-Clopper method, and the Cochran-Mantel-Haenszel test was used to compare exploratory between the two treatment arms with strata of age and previous experience with CINV. Logistic regression analyses with the backward elimination method, while keeping established prognostic factors age and previous experience of CINV fixed, were performed to determine the risk factors associated with non-achievement CR, non-achievement CC, and non-achievement TC in the overall phase and extended-overall phase. All potential explanatory variables reported in previous studies were included as independent variables. These were patient-related risk factors such as age, Eastern Cooperative Oncology Group performance status, motion sickness, habitual alcohol consumption, morning sickness, previous experience with CINV, and NK1RA use 13-16. TTF was estimated using the Kaplan-Meier method, and the curves were compared with the log-rank test and a Cox proportional hazards model. All analyses were performed using SAS version 9.4 (SAS Institute Inc., Cary, NC, USA). All P-values were two-sided, and P < 0.05 was considered statistically significant.

## Results

### Study patients

**Figure 1:**
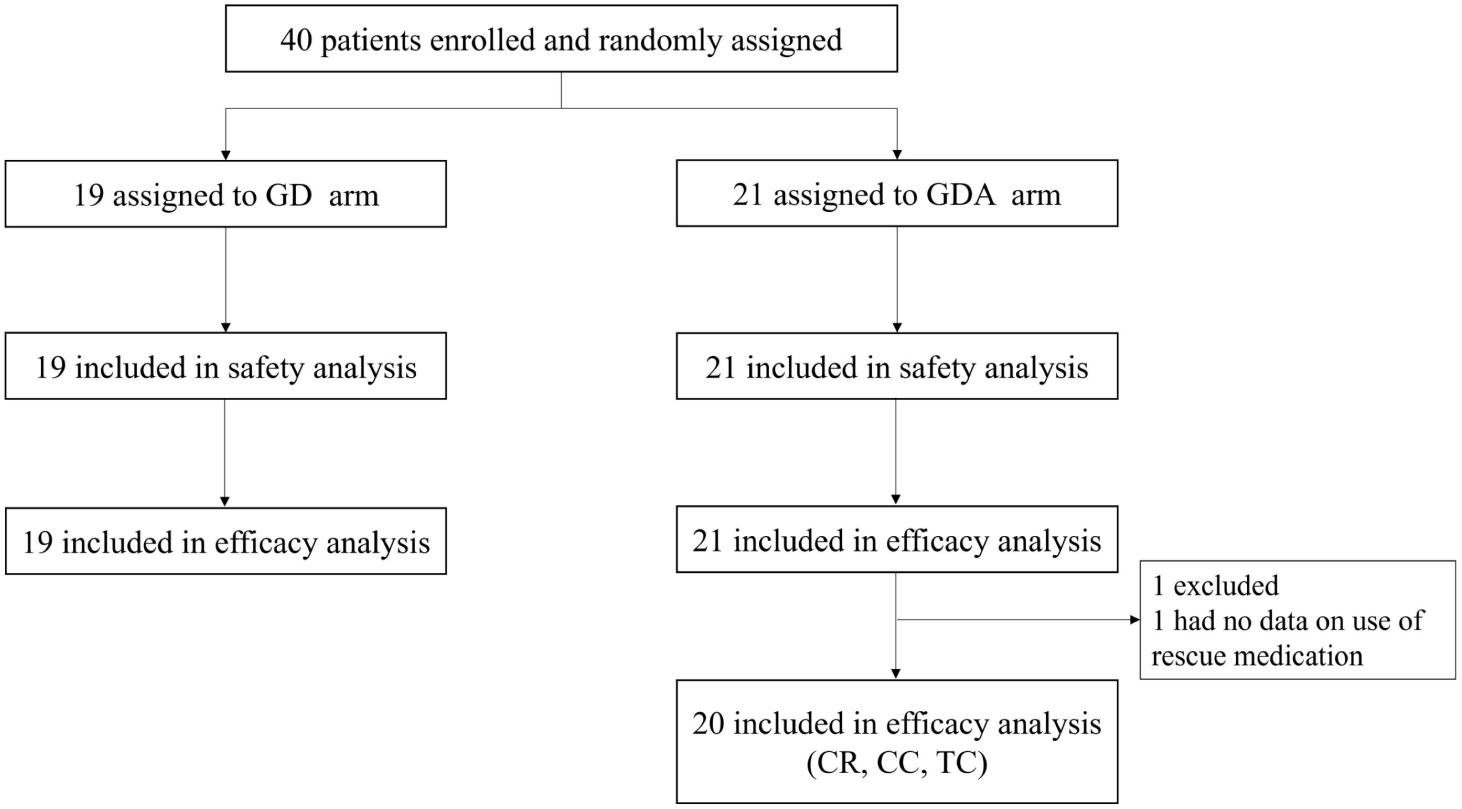
** Trial profile.** GD: patients on two-drug combination of granisetron and dexamethasone (DEX); GDA: patients on three-drug combination of granisetron, DEX, and aprepitant (fosaprepitant) CR: complete response; CC: complete control; TC: total control.

A total of 40 patients were enrolled in this study between September 2020 and March 2023. Forty patients were randomly assigned to the GD (n = 19) or GDA (n = 21) arm. In the GDA arm, one patient who did not complete the use of rescue medications, as recorded in their diary, was excluded from efficacy analysis (CR, CC, and TC rates; shown in Figure 1). Demographic data and patient characteristics are summarized in Table 1.

### Efficacy

**Figure 2:**
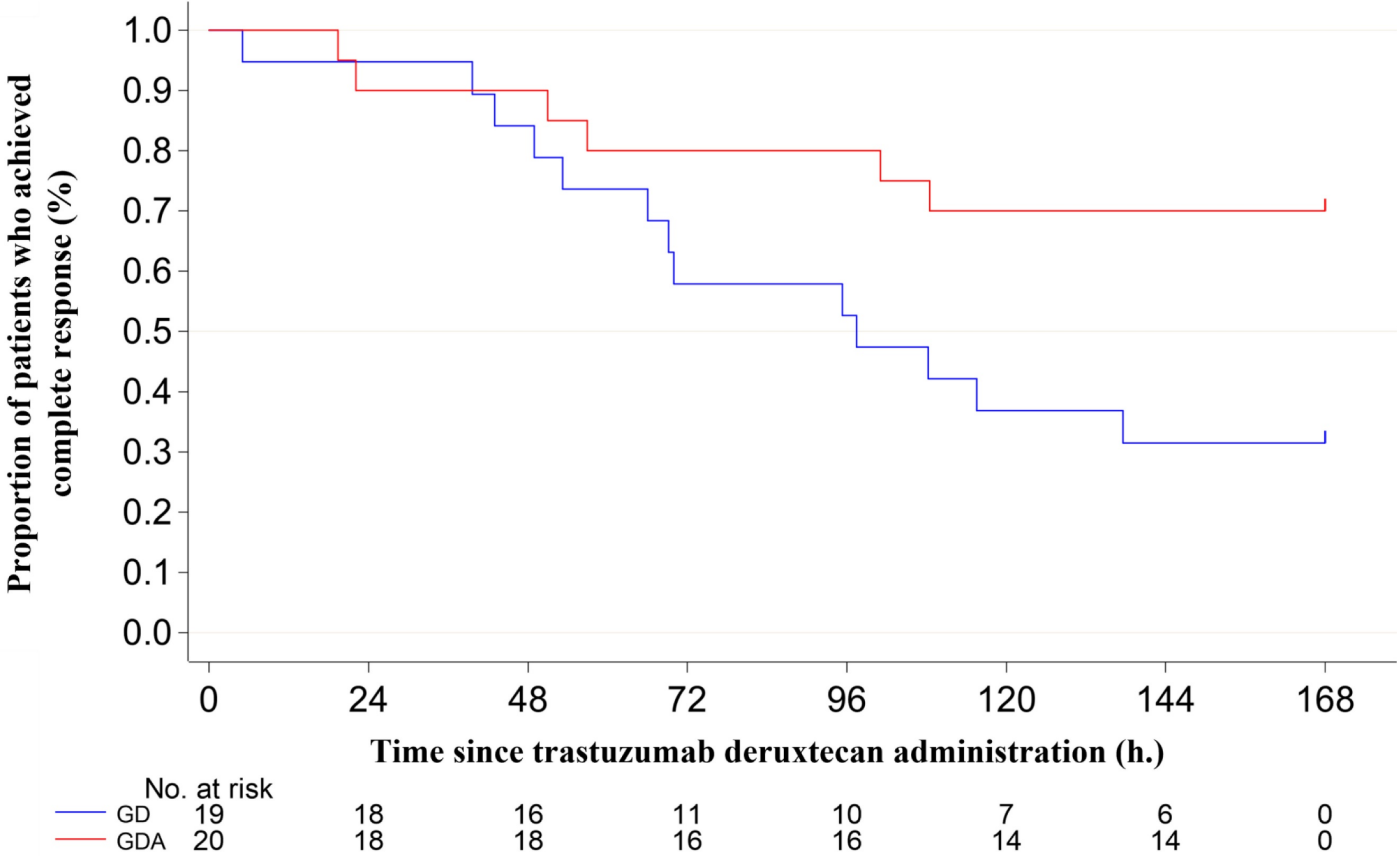
Incidence of nausea, significant nausea, and vomiting over 7 days starting on day 1 of trastuzumab deruxtecan therapy. (A) nausea, (B) significant nausea, and (C) vomiting. GD: patients on two-drug combination of granisetron and dexamethasone (DEX); GDA: patients on three-drug combination of granisetron, DEX, and aprepitant (fosaprepitant)

**Figure 3:**
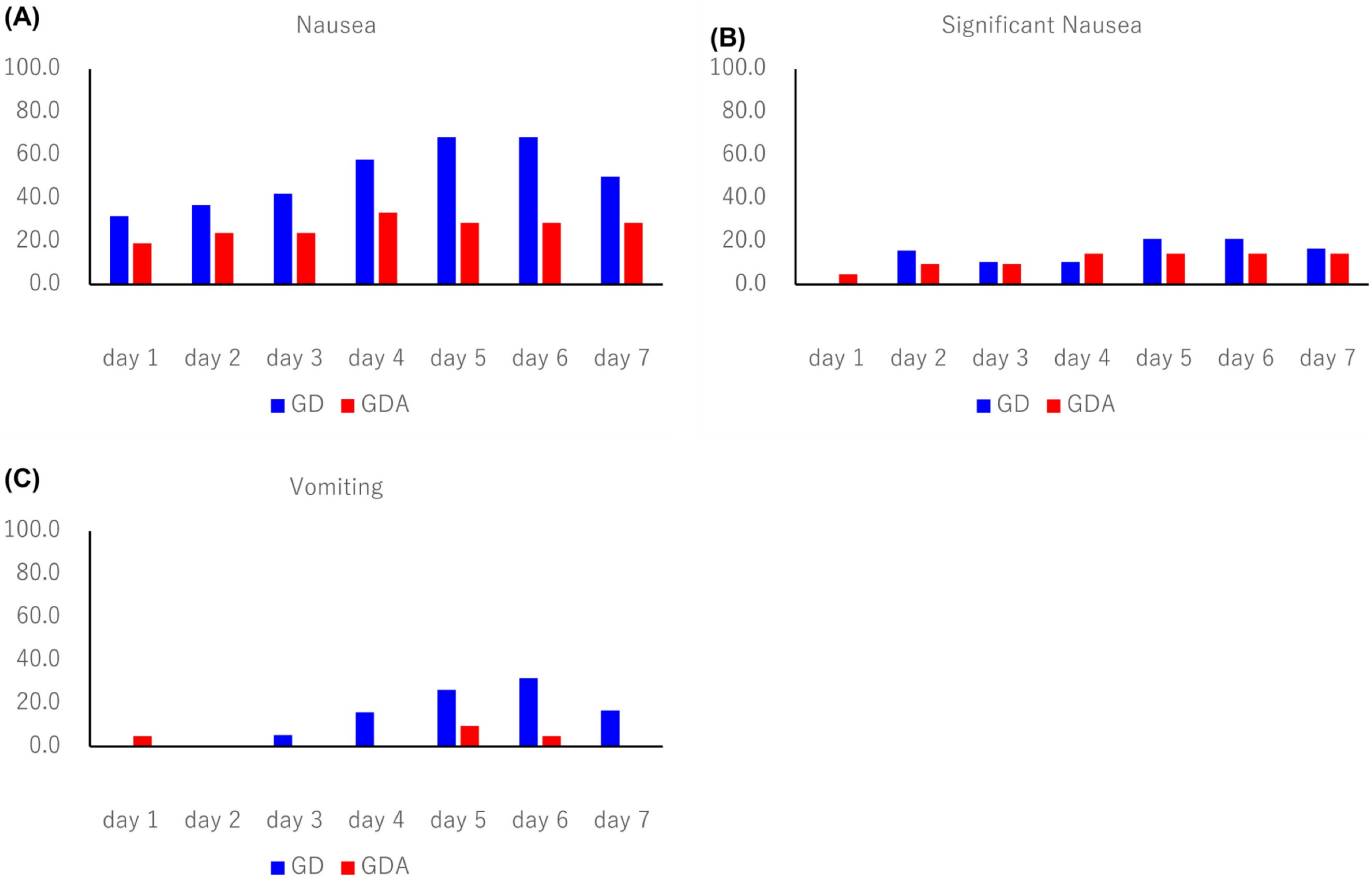
Kaplan-Meier plot showing the time to treatment failure. GD: patients on two-drug combination of granisetron and dexamethasone (DEX); GDA: patients on three-drug combination of granisetron, DEX, and aprepitant (fosaprepitant)

The antiemetic effects are summarized in Table 2. The CR rates during the overall phase were 36.8% in the GD arm and 70.0% in the GDA arm (odds ratio [OR]: 0.1334; 95% confidence interval [CI]: 0.0232-0.7672; P = 0.0190), with a difference of 33.2%. The CR rates during the extended-overall phase were 31.6% in the GD arm and 70.0% in the GDA arm (OR: 0.1073; 95% CI: 0.0185-0.6239; P = 0.0087), with a difference of 38.4%. In one patient in the GDA arm who was excluded from the evaluation of CR, CC, and TC because the rescue medication diary was not completed, nausea or vomiting did not occur during the 7-day period. The incidence of nausea, significant nausea, and vomiting after trastuzumab deruxtecan administration (shown in Figure 2) tended to increase after day 4 in both groups. The Kaplan-Meier analysis of the TTF is shown in Figure 3. TTF was significantly longer in the GDA arm than in the GD arm (hazard ratio: 0.346; 95% CI: 0.13-0.914; log-rank test, P = 0.0251).

### Adverse events

Major adverse events are summarized in Table 3. According to the investigator's evaluation, there was no grade 3 or 4 toxicity related to antiemetic therapy.

### Risks analysis

Logistic regression analysis revealed that NK1RA use was associated with a significant decrease in the non-achievement of CR, CC, and TC in overall phase and extended-overall phases (Table 4).

### Patient satisfaction

Patient satisfaction is summarized in Table 5. Only patients in the GDA arm chose to report being “very satisfied” with the treatment.

## Discussion

To our knowledge, this exploratory phase 2 study is the first to evaluate the antiemetic effects of a two-drug combination of granisetron and DEX and a three-drug combination of granisetron, DEX, and aprepitant (fosaprepitant) in trastuzumab deruxtecan-treated patients with breast cancer. Notably, the CR rates for the overall phase were 36.8% in the GD arm and 70.0% in the GDA arm, with a difference of 33.2% attributable to the addition of NK1RA. In this study, the use of the combination of 5-HT3RA and DEX with NK1RA for controlling CINV in trastuzumab deruxtecan-treated breast cancer patients resulted in a clinically meaningful difference. Additionally, multivariate analysis revealed that the addition of NK1RA significantly reduced the risk of non-achievement of CR. The Multinational Association for Supportive Care in Cancer/European Society for Medical Oncology 2016 guidelines call for a >10% improvement in CR rates on adding drugs for the prevention of CINV 11. Although the comparison between the GD and GDA arms in this study was secondary, the results met this criterion of a >10% difference.

Our study suggests that a combination of a 5-HT3RA, DEX, and an NK1RA may be more effective than that of a 5-HT3RA and DEX for the prevention of nausea and vomiting associated with trastuzumab deruxtecan. This finding is consistent with those of previous research indicating that adding an NK1RA to standard antiemetic therapy is effective and safe in preventing CINV in patients treated with highly emetogenic and carboplatin-based moderately emetogenic chemotherapy. This is because carboplatin is considered highly emetogenic even among moderately emetogenic agents 17,18.

The mechanisms underlying the high incidence of nausea and vomiting associated with trastuzumab deruxtecan treatment are not fully understood. It is possible that prolonged exposure to trastuzumab deruxtecan, which has a half-life of over six days, may contribute to these side effects 3. Extended exposure to trastuzumab deruxtecan may result in the activation of the emetic pathway, leading to nausea and vomiting. The long half-life of trastuzumab deruxtecan may be related to the high incidence of CINV observed at the 168-h time point in this study. This suggests that the prolonged presence of the drug in the body could contribute to persistent CINV symptoms. The NCCN Clinical Practice Guidelines in Oncology for Antiemetics, version 1.2023, have updated the emetic potential of trastuzumab deruxtecan from moderate to high risk. Our results support this change 9.

The present study had several limitations. First, this was an exploratory phase 2 study with a small number of patients, and an open label design, which cannot rule out the possibility of a placebo effect, particularly in patients receiving aprepitant in the GDA arm. Second, this study focused only on Japanese patients, although the results may be applicable to patients of other ethnicities. Third, our 0-168 h observation period is longer than the standard 0-124 h period for evaluating antiemetic therapy. However, trastuzumab deruxtecan, which has a half-life of more than 6 days, will require a longer observation period than 168 h in the future 19. Finally, at the start of the study, trastuzumab deruxtecan was only indicated for breast cancer in Japan despite its use in the management of a wide range of diseases; hence, the study only targeted patients with breast cancer.

Although trastuzumab deruxtecan has shown promise in the treatment of certain types of cancer, its use is associated with considerable nausea and vomiting. Unfortunately, current antiemetic regimens, including 5-HT3RAs, DEX, and NK1RAs, appear to be insufficient for controlling CINV. Therefore, it is imperative that future studies be conducted to better understand the underlying mechanisms of trastuzumab deruxtecan-induced nausea and vomiting and to develop more effective treatment strategies to manage these adverse effects in patients receiving this therapy.

## Conclusion

Breast cancer patients receiving trastuzumab deruxtecan require triple therapy, including mandatory NK1RA administration, for antiemetic prophylaxis. Future investigations are warranted to explore the development of a four-drug combination therapy including olanzapine.

## Figures and Tables

**Table 1:** Patients' characteristics

Characteristic		GD (N = 19) n (%)	GDA (N = 21) n (%)
Age	Mean ± SD	59.1 ± 11.3	60.1 ± 10.2
	Min - Max	41.0 - 77.0	41.0 - 75.0
	Median	58	60
	< 55	7 ( 36.8)	7 ( 33.3)
	>= 55	12 ( 63.2)	14 ( 66.7)
Trastuzumab deruxtecan dose (mg/kg)	Mean ± SD	5.3 ± 0.2	5.4 ± 0.0
	Min - Max	4.5 - 5.4	5.3 - 5.4
	Median	5.4	5.4
ECOG performance status	0	16 ( 84.2)	14 ( 66.7)
	1	3 ( 15.8)	7 ( 33.3)
Previous lines of therapy for metastatic disease	2	2 ( 10.5)	3 ( 14.3)
	3	8 ( 42.1)	8 ( 38.1)
	4	3 ( 15.8)	3 ( 14.3)
	6	3 ( 15.8)	3 ( 14.3)
	7	0 ( 0.0)	1 ( 4.8)
	8	1 ( 5.3)	1 ( 4.8)
	9	1 ( 5.3)	1 ( 4.8)
	10	1 ( 5.3)	0 ( 0.0)
	11 or more	0 ( 0.0)	1 ( 4.8)
Motion sickness	No	14 ( 73.7)	19 ( 90.5)
	Yes	5 ( 26.3)	2 ( 9.5)
Habitual alcohol consumption	No	18 ( 94.7)	15 ( 71.4)
	Yes	1 ( 5.3)	6 ( 28.6)
Morning sickness	No	5 ( 26.3)	8 ( 38.1)
	Yes	11 ( 57.9)	9 ( 42.9)
	no experience	3 ( 15.8)	4 ( 19.0)
Previous experience of nausea and vomiting with chemotherapy	No	9 ( 47.4)	11 ( 52.4)
	Yes	10 ( 52.6)	10 ( 47.6)

**Table 2:** Control of CINV

Endpoints	GD (N = 19)		GDA (N = 20)	
	No.	(%)	No.	(%)
Complete response				
Overall phase	7	36.8	14	70.0
Extended-overall phase	6	31.6	14	70.0
Acute phase	18	94.7	18	90.0
Delayed phase	7	36.8	15	75.0
Extended-delayed phase	6	31.6	14	70.0
Complete control				
Overall phase	5	26.3	12	60.0
Extended-overall phase	4	21.1	12	60.0
Acute phase	18	97.4	17	85.0
Delayed phase	5	26.3	13	65.0
Extended-delayed phase	4	21.1	13	65.0
Total control				
Overall phase	2	10.5	9	45.0
Extended-overall phase	2	10.5	8	40.0
Acute phase	13	68.4	14	70.0
Delayed phase	2	10.5	11	55.0
Extended-delayed phase	2	10.5	9	45.0
**Endpoints**	**GD (N = 19)**		**GDA (N = 21)**	
	**No.**	**(%)**	**No.**	**(%)**
No nausea				
Overall phase	3	15.8	12	57.1
Extended-overall phase	2	10.5	11	52.4
Acute phase	13	68.4	17	81.0
Delayed phase	4	21.1	13	61.9
Extended-delayed phase	3	15.8	12	57.1
No significant nausea				
Overall phase	14	73.7	18	85.7
Extended-overall phase	13	68.4	18	85.7
Acute phase	19	100.0	20	95.2
Delayed phase	14	73.7	18	85.7
Extended-delayed phase	13	68.4	18	85.7
No vomiting				
Overall phase	13	68.4	18	85.7
Extended-overall phase	10	52.6	17	81.0
Acute phase	19	100.0	20	95.2
Delayed phase	13	68.4	19	90.5
Extended-delayed phase	10	52.6	18	85.7

**Table 3:** Adverse events

Symptom Term	GD (N=19) n (%)					GDA (N=21) n (%)				
	Grade1	Grade2	Grade3	Grade4	Unknown	Grade1	Grade2	Grade3	Grade4	Unknown
Nausea	9 ( 47.4)	6 ( 31.6)	0 ( 0.0)	0 ( 0.0)	1 ( 5.3)	8 ( 38.1)	2 ( 9.5)	0 ( 0.0)	0 ( 0.0)	0 ( 0.0)
Vomiting	4 ( 21.1)	0 ( 0.0)	0 ( 0.0)	0 ( 0.0)	1 ( 5.3)	1 ( 4.8)	0 ( 0.0)	0 ( 0.0)	0 ( 0.0)	0 ( 0.0)
Anorexia	9 ( 47.4)	6 ( 31.6)	1 ( 5.3)	0 ( 0.0)	1 ( 5.3)	10 ( 47.6)	3 ( 14.3)	0 ( 0.0)	0 ( 0.0)	0 ( 0.0)
Dysgeusia	6 ( 31.6)	1 ( 5.3)	0 ( 0.0)	0 ( 0.0)	1 ( 5.3)	2 ( 9.5)	1 ( 4.8)	0 ( 0.0)	0 ( 0.0)	0 ( 0.0)
Hiccups	2 ( 10.5)	0 ( 0.0)	0 ( 0.0)	0 ( 0.0)	1 ( 5.3)	1 ( 4.8)	0 ( 0.0)	0 ( 0.0)	0 ( 0.0)	0 ( 0.0)
Constipation	7 ( 36.8)	2 ( 10.5)	0 ( 0.0)	0 ( 0.0)	1 ( 5.3)	9 ( 42.9)	2 ( 9.5)	0 ( 0.0)	0 ( 0.0)	0 ( 0.0)
Diarrhea	3 ( 15.8)	0 ( 0.0)	0 ( 0.0)	0 ( 0.0)	1 ( 5.3)	2 ( 9.5)	0 ( 0.0)	0 ( 0.0)	0 ( 0.0)	0 ( 0.0)
**Symptom Term (PRO-CTCAE/CTCAE)**	**GD (N=19)** **n (%)**					**GDA (N=21)** **n (%)**				
	**Mild**	**Moderate**	**Severe**	**Very severe**	**Unknown**	**Mild**	**Moderate**	**Severe**	**Very severe**	**Unknown**
Nausea (severity)	6 ( 31.6)	6 ( 31.6)	1 ( 5.3)	0 ( 0.0)	1 ( 5.3)	7 ( 33.3)	1 ( 4.8)	2 ( 9.5)	0 ( 0.0)	0 ( 0.0)
Nausea (interference)	5 ( 26.3)	9 ( 47.4)	2 ( 10.5)	0 ( 0.0)	1 ( 5.3)	3 ( 14.3)	4 ( 19.0)	2 ( 9.5)	2 ( 9.5)	0 ( 0.0)
Vomiting (severity)	1 ( 5.3)	2 ( 10.5)	1 ( 5.3)	0 ( 0.0)	1 ( 5.3)	2 ( 9.5)	0 ( 0.0)	0 ( 0.0)	1 ( 4.8)	0 ( 0.0)
Vomiting (interference)	0 ( 0.0)	4 ( 21.1)	0 ( 0.0)	0 ( 0.0)	1 ( 5.3)	1 ( 4.8)	0 ( 0.0)	0 ( 0.0)	0 ( 0.0)	0 ( 0.0)
Anorexia (severity)	3 ( 15.8)	9 ( 47.4)	4 ( 21.1)	0 ( 0.0)	1 ( 5.3)	6 ( 28.6)	5 ( 23.8)	1 ( 4.8)	1 ( 4.8)	1 ( 4.8)
Anorexia (interference)	7 ( 36.8)	5 ( 26.3)	6 ( 31.6)	0 ( 0.0)	1 ( 5.3)	8 ( 38.1)	4 ( 19.0)	2 ( 9.5)	2 ( 9.5)	0 ( 0.0)
Taste changes/Dysgeusia	6 ( 31.6)	3 ( 15.8)	1 ( 5.3)	1 ( 5.3)	1 ( 5.3)	1 ( 4.8)	4 ( 19.0)	1 ( 4.8)	0 ( 0.0)	0 ( 0.0)
Hiccups (severity)	2 ( 10.5)	0 ( 0.0)	0 ( 0.0)	0 ( 0.0)	1 ( 5.3)	3 ( 14.3)	0 ( 0.0)	0 ( 0.0)	0 ( 0.0)	0 ( 0.0)
Hiccups (interference)	0 ( 0.0)	2 ( 10.5)	0 ( 0.0)	0 ( 0.0)	1 ( 5.3)	4 ( 19.0)	1 ( 4.8)	0 ( 0.0)	0 ( 0.0)	0 ( 0.0)
Constipation	2 ( 10.5)	9 ( 47.4)	4 ( 21.1)	0 ( 0.0)	1 ( 5.3)	5 ( 23.8)	7 ( 33.3)	2 ( 9.5)	0 ( 0.0)	0 ( 0.0)
Diarrhea	3 ( 15.8)	2 ( 10.5)	0 ( 0.0)	0 ( 0.0)	1 ( 5.3)	2 ( 9.5)	3 ( 14.3)	1 ( 4.8)	0 ( 0.0)	1 ( 4.8)

**Table 4:** Risk analysis for CINV in overall phase and extended-overall phase

Non-achievement of CR (overall phase)	Univariate analysis		Multivariate analysis	
	OR (95%CI)	*P* value	OR (95%CI)	*P* value
Age (< vs. >=55 years)	1.273 (0.343 - 4.726)	0.7186	0.996 (0.227 - 4.371)	0.9957
PS (1 vs. 0)	1.714 (0.329 - 8.943)	0.5225		
Motion sickness (Yes vs. No)	0.203 (0.037 - 1.127)	0.0683		
Habitual alcohol consumption (Yes vs. No)	1.714 (0.329 - 8.943)	0.5225		
Morning sickness (Yes vs. No)	0.714 (0.169 - 3.027)	0.6479		
Morning sickness (No experience vs. No)	0.952 (0.144 - 6.281)	0.9596		
Previous experience of nausea and vomiting with chemotherapy (Yes vs. No)	2.095 (0.581 - 7.555)	0.2583	2.215 (0.529 - 9.284)	0.2767
With APR (GDA vs. GD)	0.250 (0.066 - 0.950)	0.0419	0.242 (0.062 - 0.950)	0.0420
**Non-achievement CC (overall phase)**	**Univariate analysis**		**Multivariate analysis**	
	**OR (95%CI)**	***P* value**	**OR (95%CI)**	***P* value**
Age (< vs. >=55 years)	2.708 (0.668 - 10.98)	0.1631	2.565 (0.540 - 12.19)	0.2363
PS (1 vs. 0)	1.037 (0.199 - 5.410)	0.9656		
Motion sickness (Yes vs. No)	0.226 (0.048 - 1.067)	0.0604		
Habitual alcohol consumption (Yes vs. No)	2.206 (0.372 - 13.09)	0.3839		
Morning sickness (Yes vs. No)	1.500 (0.355 - 6.347)	0.5817		
Morning sickness (No experience vs. No)	1.333 (0.204 - 8.708)	0.7638		
Previous experience of nausea and vomiting with chemotherapy (Yes vs. No)	2.063 (0.570 - 7.470)	0.2698	1.709 (0.402 - 7.275)	0.4682
With APR (GDA vs. GD)	0.238 (0.061 - 0.925)	0.0383	0.220 (0.053 - 0.913)	0.0370
**Non-achievement TC (overall phase)**	**Univariate analysis**		**Multivariate analysis**	
	**OR (95%CI)**	***P* value**	**OR (95%CI)**	***P* value**
Age (< vs. >=55 years)	1.725 (0.374 - 7.953)	0.4844	1.397 (0.245 - 7.952)	0.7066
PS (1 vs. 0)	2.727 (0.289 - 25.75)	0.3811		
Motion sickness (Yes vs. No)	0.261 (0.056 - 1.206)	0.0854		
Habitual alcohol consumption (Yes vs. No)	0.978 (0.160 - 5.988)	0.9810		
Morning sickness (Yes vs. No)	0.467 (0.078 - 2.807)	0.4051		
Morning sickness (No experience vs. No)	0.267 (0.032 - 2.249)	0.2243		
Previous experience of nausea and vomiting with chemotherapy (Yes vs. No)	2.333 (0.554 - 9.834)	0.2483	2.340 (0.456 - 12.00)	0.3080
With APR (GDA vs. GD)	0.144 (0.026 - 0.795)	0.0262	0.136 (0.024 - 0.784)	0.0256
**Non-achievement CR (extended-overall phase)**	**Univariate analysis**		**Multivariate analysis**	
	**OR (95%CI)**	***P* value**	**OR (95%CI)**	***P* value**
Age (< vs. >=55 years)	1.083 (0.293 - 4.011)	0.9046	0.877 (0.196 - 3.935)	0.8641
PS (1 vs. 0)	1.511 (0.290 - 7.869)	0.6239		
Motion sickness (Yes vs. No)	0.176 (0.032 - 0.982)	0.0477		
Habitual alcohol consumption (Yes vs. No)	1.511 (0.290 - 7.869)	0.6239		
Morning sickness (Yes vs. No)	1.711 (0.403 - 7.271)	0.4668		
Morning sickness (No experience vs. No)	1.050 (0.159 - 6.924)	0.9596		
Previous experience of nausea and vomiting with chemotherapy (Yes vs. No)	1.681 (0.473 - 5.967)	0.4220	1.823 (0.429 - 7.748)	0.4160
With APR (GDA vs. GD)	0.198 (0.051 - 0.771)	0.0195	0.194 (0.049 - 0.770)	0.0197
**Non-achievement CC (extended-overall phase)**	**Univariate analysis**		**Multivariate analysis**	
	**OR (95%CI)**	***P* value**	**OR (95%CI)**	***P* value**
Age (< vs. >=55 years)	2.308 (0.569 - 9.359)	0.2417	2.348 (0.476 - 11.57)	0.2944
PS (1 vs. 0)	0.912 (0.174 - 4.773)	0.9134		
Motion sickness (Yes vs. No)	0.193 (0.040 - 0.922)	0.0393		
Habitual alcohol consumption (Yes vs. No)	1.944 (0.327 - 11.55)	0.4648		
Morning sickness (Yes vs. No)	1.857 (0.432 - 7.978)	0.4052		
Morning sickness (No experience vs. No)	1.333 (0.204 - 8.708)	0.7638		
Previous experience of nausea and vomiting with chemotherapy (Yes vs. No)	1.671 (0.462 - 6.051)	0.4339	1.389 (0.315 - 6.122)	0.6642
With APR (GDA vs. GD)	0.178 (0.043 - 0.736)	0.0171	0.167 (0.039 - 0.724)	0.0168
**Non-achievement TC (extended-overall phase)**	**Univariate analysis**		**Multivariate analysis**	
	**OR (95%CI)**	***P* value**	**OR (95%CI)**	***P* value**
Age (< vs. >=55 years)	1.426 (0.304 - 6.695)	0.6530	1.191 (0.212 - 6.701)	0.8424
PS (1 vs. 0)	2.348 (0.247 - 22.34)	0.4578		
Motion sickness (Yes vs. No)	0.208 (0.043 - 1.001)	0.0502		
Habitual alcohol consumption (Yes vs. No)	0.833 (0.134 - 5.167)	0.8447		
Morning sickness (Yes vs. No)	0.212 (0.022 - 2.032)	0.1786		
Morning sickness (No experience vs. No)	0.121 (0.010 - 1.531)	0.1029		
Previous experience of nausea and vomiting with chemotherapy (Yes vs. No)	1.846 (0.428 - 7.961)	0.4111	1.814 (0.356 - 9.245)	0.4733
With APR (GDA vs. GD)	0.177 (0.032 - 0.982)	0.0477	0.174 (0.031 - 0.985)	0.0480

Abbreviations, CR: complete response; CC: complete control; TC: total control

**Table 5:** Patient satisfaction

	GD (N = 19) n (%)	GDA (N = 21) n (%)
Very satisfied	0 ( 0.0)	7 ( 33.3)
Satisfied	8 ( 42.1)	5 ( 23.8)
Somewhat satisfied	4 ( 21.1)	2 ( 9.5)
Rather satisfied	2 ( 10.5)	2 ( 9.5)
Rather dissatisfied	2 ( 10.5)	1 ( 4.8)
Dissatisfied	1 ( 5.3)	1 ( 4.8)
Very dissatisfied	1 ( 5.3)	1 ( 4.8)
Blank	1 ( 5.3)	2 ( 9.5)

## Abbreviations

CC: complete control
CI: confidence interval
CINV: chemotherapy-induced nausea and vomiting
CR: complete response
CTCAE: Common Terminology Criteria for Adverse Events
DEX: dexamethasone
5-HT3RA: 5-hydroxytryptamine-3 receptor antagonist
GD: granisetron and dexamethasone
GDA: granisetron, dexamethasone, and aprepitant (fosaprepitant)
HER2: human epidermal growth factor receptor 2
NCCN: National Comprehensive Cancer Network
NK1RA: neurokinin-1 receptor antagonist
OR: odds ratio
PRO: patient-reported outcome
TC: total control
TTF: time to treatment failure
